# mRNA stability and the unfolding of gene expression in the long-period yeast metabolic cycle

**DOI:** 10.1186/1752-0509-3-18

**Published:** 2009-02-06

**Authors:** Nicola Soranzo, Mattia Zampieri, Lorenzo Farina, Claudio Altafini

**Affiliations:** 1 SISSA, via Beirut 4, 34014 Trieste, Italy; 2 Università degli Studi di Roma "La Sapienza", via Ariosto 25, 00185 Roma, Italy

## Abstract

**Background:**

In yeast, genome-wide periodic patterns associated with energy-metabolic oscillations have been shown recently for both short (approx. 40 min) and long (approx. 300 min) periods.

**Results:**

The dynamical regulation due to mRNA stability is found to be an important aspect of the genome-wide coordination of the long-period yeast metabolic cycle. It is shown that for periodic genes, arranged in classes according either to expression profile or to function, the pulses of mRNA abundance have phase and width which are directly proportional to the corresponding turnover rates.

**Conclusion:**

The cascade of events occurring during the yeast metabolic cycle (and their correlation with mRNA turnover) reflects to a large extent the gene expression program observable in other dynamical contexts such as the response to stresses/stimuli.

## Background

Ultradian self-sustaining energy-metabolic oscillations arising spontaneously in high density *Saccharomyces cerevisiae *continuous cultures exposed to glucose-limited growth have been known and studied for decades [1,2], and have more recently been observed to induce genome-wide periodic patterns in different series of microarray experiments [3,4], although with widely different periodicities, ~40 min for [3] and ~300 min for [4].

Many studies aim at understanding the mechanisms inducing these sustained oscillations and the rigorous temporal compartmentalization they induce, see [5,6] for surveys. Suggested causes range from a single critical pathway (like the feedback effect of cysteine on the sulfur assimilation pathway [7]) to the alternation of aerobic and anaerobic respiratory modes (as deduced by the fluctuations in the concentration of dissolved *O*_2 _and of other observed metabolites [4]), from the interaction with cell cycle [8,9] to the mutual incompatibility of different redox biochemical processes [10,11].

The scope of this work is to emphasize a different aspect, intrinsically dynamical and post-transcriptional, which is likely to play an important role in the coordination of the "slower" yeast metabolic cycle (YMC) of [4], namely mRNA stability. We will show that there is a roughly linear relationship between the average half life (HL) of the transcripts, clustered according to expression or function, and the phase at which their concentration peaks in the cycle. More generally, there seems to be a strong correlation between HL and the shape of the pulses of gene expression: genes with short HL have short and sharp (almost impulsive in the time scale considered) pulses, while genes with long HL have pulses that are not only delayed but also broader and with more gentle slopes.

In recent years, post-transcriptional control is being recognized as an important aspect of gene regulation, especially in eukaryotic DNA, which lacks operonal structure [12-15]. It can occur in many guises, through mRNA turnover [16-20], or through "RNA regulons" [21] i.e., groups of genes coordinately guided in the RNA processing, localization and protein synthesis by RNA-binding proteins (RBPs) [22,23], or even through the mediation of a metabolic substrate (typically a nutrient [24-26] or an enzyme [27]). Our result confirms the importance of post-transcriptional control, and points at mRNA turnover as a regulatory mechanism at a genome-wide level. Its peculiarity consists in putting the time axis into the picture in an intrinsically dynamical way. Consequently, in order to be observed, it requires times series sampled at a sufficiently high frequency and dynamics in the right time window, a combination seldom occurring in current expression profiling datasets. So for example the correlation between HL and phase/shape of the oscillations cannot be observed in the much faster YMC of [3], where HL and the period are of comparable duration, hence the system has no time to decay before the arrival of the next wavefront.

In order to emphasize the dynamical aspects, we shall treat the YMC as the time response of a genome-wide dynamical system to a sequence of impulsive "inputs" of transcription activation. We will show that grouping genes in terms of progressively delayed and broadened responses to a sequence of "input pulses" of transcriptional activation allows to see in a remarkably fine detail the causal chain of events constituting the transcriptional program of the cell. The few ambiguities resulting from this classification can be interpreted in terms of some other annotation, typically compartmental localization.

In the following we shall proceed in two complementary ways: first the YMC time series are clustered in a completely unsupervised manner, only according to gene expression. The linear relationship between pulse phase (also pulse width) and HL then emerges in a straightforward way. Next, we consider families of genes whose products share some common annotation, for example genes on the same pathway or genes that are subunits of the same protein complex, and look at the type of time series they produce and at their "position" along the YMC.

Both approaches confirm that the YMC represents an organized cascade of events, in response to precisely equispaced bursts of transcriptional activation, with the temporal order reflecting the transcript turnover rate. Extrapolating from the specific YMC context, this cascade of events is observable to a good extent also in other gene expression time series (such as the response to a pulse of nutrient of [28], or the stress responses of [29]), suggesting it might reflect a prototypical dynamical mode of action of transcriptional response.

## Results and discussion

**Figure 1:**
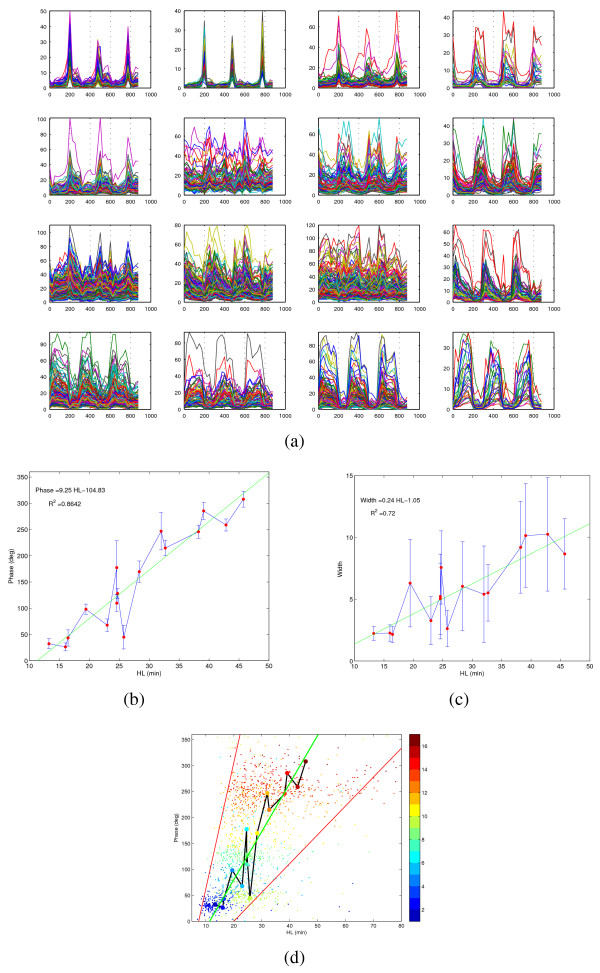
**Linearity of the relation phase/HL in the clustered YMC**. In (a) the time series (on the x axis: time in min.) of the periodic genes is clustered according to a nonnormalized correlation distance function (see Table 1 for details on the clusters). The clusters are then sorted (from left to right from top to bottom) according to the average HL. Moving along the clusters, a change in the phase and in the width of the pulses is clearly visible, thus suggesting a direct relationship between HL and phase/width of the pulses. This is made explicit in (b), where the average HL is plotted against the average phase for each cluster, and in (c) where the average HL is shown against the average pulse width. In the scatter plot of HL versus phase (d), the color indicates the cluster number (see colorbar on the right). As can be noticed, along the HL axis the standard deviation of a cluster grows with the mean, see Table 1 for exact values, and the cloud of points looks like a cone (the cone delimited by the two red lines contains 95% of the periodic genes). Still the increase of the phase with the HL is clearly visible. In the least-squares linear fit in (b) (green) half of the *L*_2 _norm of the residues is due to Cluster 9 (cytoplasmic ribosomes, see text). The p. value for both linear regressions is < 10^-5^. Further details on these regressions are provided in Additional file 1. It is worth remarking that the direct proportionality phase/HL is robust with respect to the number of clusters chosen.

**Figure 2:**
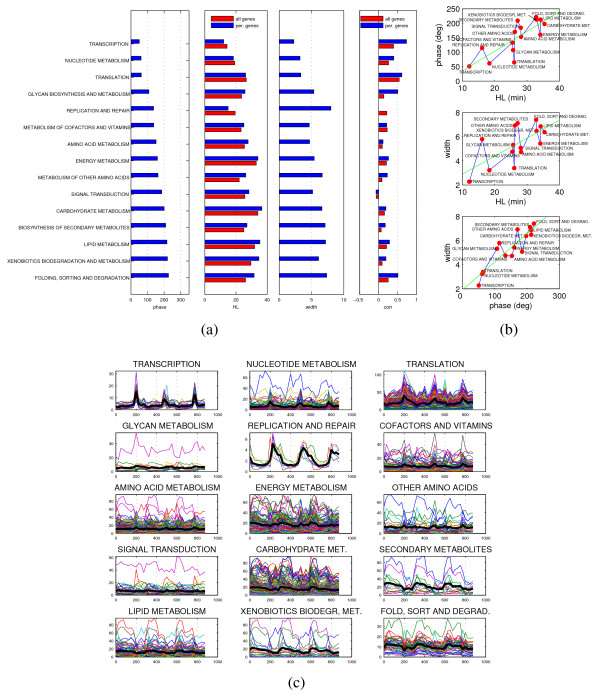
**Gene expression program emerging from the YMC**. (a): The periodic genes of the YMC are grouped according to KEGG pathways (see Fig. S2 in Additional file 1) and then in the 15 macrocategories shown. For each macrocategory we calculate the average phase, HL, pulse width, and correlation of the periodic genes (in blue), and also the average HL and correlation of all genes (in red). Sorting by phase reveals the expected concatenation of events of the yeast gene expression program, especially in the first part with transcription preceding protein synthesis and DNA replication, followed by the slower categories of central metabolism. (b): Comparing HL and phase (or pulse width) roughly the same type of direct proportionality still appear. The trend in the average profiles of each category (black thick lines in (c)) reflects to a large extent that of Fig. 1. The third plot in (b) shows that also phase and pulse width are directly correlated: pulses that are delayed are also broadened. Linear regression for these plots is discussed in Additional file 1.

**Figure 3:**
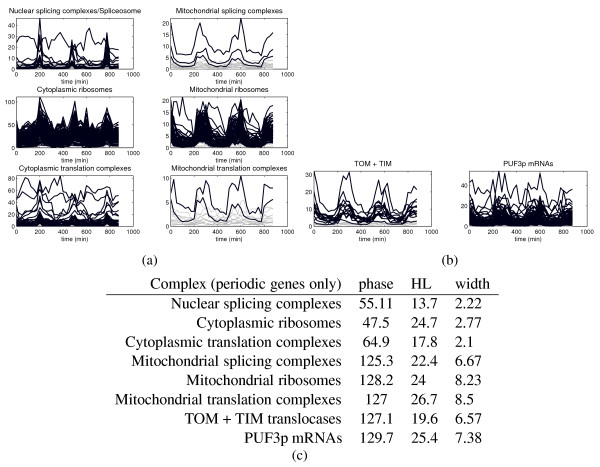
**Mitochondrial compartmentalization**. (a): Cytoplasmic vs. mitochondrial splicing, ribosomal (small and large subunits are lumped together) and ribosomal translational complexes. All genes are nuclear-encoded. Black profiles represent mRNAs classified as periodic. Within each of the two compartments, the time courses of gene expression are similar and fairly coordinated. Even the amount of correlation among the complexes subunits is similar, with e.g. ribosomal mRNAs in both compartments being more tightly coordinated than the corresponding translational machineries. The bursts for the cytoplasmic localizations are much sharper, higher and shorter than in the mitochondria. These last accumulate an average phase lag of ~90^°^, or around 50 minutes of delay (recall that the phase is computed by autocorrelation with a train of sinusoids, hence the value for the phase represents the "center" of the pulse). The cytoplasmic ribosomal complex substantially overlaps with cluster 9 of Fig. 1(a), while the mitochondrial ribosomal complex is contained in cluster 8 of the same Figure. (b): Mitochondrial translocases across outer and inner membranes, and mRNAs having Puf3p as a RBP (220 genes, 134 periodic). Of the 236 mRNAs belonging to at least one of the mitochondrial categories shown in the Figure, 62 have Puf3p as RBP. This tells us that in this case the "localization" constraint is stronger than co-sharing a single RBP, but that the two conditions are coupled and induce a similar pattern of dynamical regulation.

The ~2000 genes labeled as periodic by a periodogram test are subdivided into 16 clusters, see Fig. 1. In Fig. 1(a) the clusters are sorted in increasing order of HL (computed as the average of the HLs of the cluster elements). It is immediately evident that the typical profiles, both in terms of the phase of the peaks (for each gene the phase is computed maximizing the correlation with respect to a train of shifted sinusoids) and of their width (although in a less regular way) is modified in an almost continuous manner as we move along the clusters figures. Notice in particular how the peaks of the first clusters match the "valleys" of the last ones. For the average phase on each cluster, the phase/HL relationship is almost linear (Fig. 1(b)). The scatter plot in (d) confirms this linear proportionality, but also shows a growing variance along the HL axis (see Table 1 for details). The deviations from linearity of clusters 6 and 9 admit a reasonable explanation, mostly in terms of compartmental localization. Cluster 6 is essentially composed of retrotransposons (all Ty1 and Ty2) and long term repeat mRNAs (mostly of *δ *type) for a total of 73 out of 102 genes. For most of these genes (59) an HL measure is missing. Hence the average HL for this cluster (and this cluster alone) may be biased or unreliable. Cluster 9 instead is almost entirely composed of cytoplasmic ribosomal subunits (109 out of 151 genes). In between, Clusters 7 and 8 contain to a large extent genes with mitochondrial localization and/or function (mitochondria organization and biogenesis, protein import into mitochondrial matrix, oxidoreductase activity for Cluster 7, mitochondrial ribosomes, envelope and membranes for Cluster 8). As is explained in detail in the next paragraph, the large deviation from linearity seen in Cluster 9 can be due to an extremely fast and short lived response of the mRNAs deputed to the biosynthesis of the cytoplasmic ribosomal complexes, not deducible from the available HL data, neither from the current literature (in [30] it is affirmed that cytoplasmic ribosomal genes tend to be stabilized by nutrient uptake). Although less precise, also the relation between HL and pulse width on each cluster (Fig. 1(c)) is approximately linear. Unlike the phase/HL proportionality, this last result is expected from simple dynamical considerations, as longer HL means longer "kernel width", see also the dynamical model explanation below. The emergence of a linear relation between HL and phase once the genes are arranged in classes according to profile similarity suggests that a corresponding cascade of causally organized events may be taking place during the YMC. To some extent this is already visible through an ontological analysis of the clusters of Fig. 1 (see Table 1), but in order to investigate more in detail the biological meaning and significance of such a genomic "assembly line" we computed HLs, phases and pulse widths along the main yeast pathways and for some of the annotated yeast protein complexes. The data for the pathways (see Fig. S2 in Additional file 1) are then lumped together into the 15 functional macrocategories shown in Fig. 2. In terms of these macrocategories (sorted by phase), the result is that the mRNAs activation reflects tightly the gene expression program expected to take place in the cell, especially for the "fast" categories, i.e., transcription, nucleotide metabolism and translation starting essentially synchronously in the time scale of the YMC, followed by DNA replication and repair and amino acid metabolism. Progressing further toward the slow processes, one encounters the metabolism of energy, carbohydrates and lipids. Also for this classification, the progression in terms of phase along the cycle is substantially faithful to the increase in HL (in the top plot of Fig. 2(b) the most significant outlier is still the category "translation" already mentioned, see also Fig. 3), and the progression in phase is paralleled by an increase in pulse width (see bottom plot of Fig. 2(b)).

**Table 1:** Statistics for the 16 clusters for Fig. 1

cl.	genes	HL		phase		width		ontology
		mean	std	mean	std	mean	std	
1	101	13.26	(9.54)	32.4	(9.4)	2.2	(0.56)	RNA, rRNA, and tRNA processing and metabolism, ribosome biogenesis and assembly
2	58	16.02	(19.07)	26.3	(7.6)	2.3	(0.66)	RNA, rRNA and tRNA processing and metabolism, RNA helicase, ribosome assembly
3	101	16.46	(8.65)	43.3	(15.4)	2.1	(0.65)	RNA polymerase, translation initiation, regulation, and termination, nucleotide biosynthesis
4	34	19.44	(10.19)	98.2	(9.7)	6.3	(3.54)	transferase activity, DNA replication, cell cycle
5	102	22.99	(10.27)	67.7	(11.8)	3.3	(1.95)	glycine metabolism, nitrogen and sulfur metabolism, amino acid biosynthesis
6	102	24.59	(11.67)	177.4	(51.0)	5.2	(3.43)	retrotransposons, long term repeats
7	124	24.59	(13.45)	109.6	(15.8)	5.0	(2.88)	mitochondrial membrane organization and biogenesis, mitochondrial transport
8	151	24.72	(11.80)	128.3	(9.4)	7.6	(2.96)	mitochondrial ribosome, envelope, and membranes
9	232	25.76	(13.78)	44.8	(22.5)	2.6	(1.46)	cytoplasmic ribosomes, translation processes
10	154	28.34	(16.36)	169.7	(20.3)	6.0	(3.59)	ion/cation transmembrane transport, electron transport, oxidative phosphorylation
11	230	31.99	(19.05)	246.7	(35.5)	5.4	(3.90)	endopeptidase activity, protein catabolic process, proteasome, actin filament organization, glycolysis, gluconeogenesis
12	65	32.69	(18.68)	214.8	(14.8)	5.5	(2.28)	lipid and alcohol metabolic process, peroxisome
13	223	38.24	(28.35)	245.8	(12.6)	9.2	(3.71)	kinase activity, vacuolar transport, membrane organization and biogenesis
14	128	39.10	(29.27)	285.5	(16.1)	10.1	(4.19)	arginine biosynthesis, protein folding
15	117	42.83	(28.02)	258.7	(11.5)	10.2	(4.59)	hydrolase activity, fatty acid oxidation, cytokinesis
16	29	45.74	(26.30)	307.8	(15.1)	8.7	(2.84)	catalytic activity

### A detailed functional analysis

Using the ordering by phase of pathways and protein complexes (see Fig. S2 and S5 in Additional file 1), we can zoom on these categories in much more detail. The first phase of this cascade consists of the activation of the transcription machinery with the synchronous bursts of transcription of the three RNA polymerases (see Fig. S1 in Additional file 1) and of most of the RNA processing components, like the tRNA processing complexes (RNase P) and rRNA processing complexes (exosome, RNase MRP, *SIK1*, *NOP1*), with the nuclear splicing complexes following closely. While the mRNAs for the polymerases are highly coordinated, the same cannot be said for the basal transcription factors (TFs) required for their initiation. Overall only a few of these genes follow the bursting trend of the RNA polymerases, notably, among them, *SPT15*, which forms the TATA-binding protein and is also a component of the polymerase I core factor and of TFIIIB. Most other genes involved with these general TFs do not show any periodic pattern, and their mRNA concentrations never surpass very low levels.

From Fig. 2, the peak of mRNA concentrations associated with the category "translation" seems to be synchronous with the RNA processing burst. However, a more careful analysis reveals that this phase is an average of two "compartmentalized" activations of the translation machinery, having fairly different phases: while cytoplasmic translation follows almost simultaneously the RNA machinery, the mitochondrial translation activation has a phase lag of more than one sixth of the period. In terms of time delay, this amounts to approximately 50 min, see Fig. 3. More in detail, most of the mRNAs of ribosomal small and large subunits for both cytoplasmic and mitochondrial localizations are highly correlated within their complex (average Pearson correlation for both is around 0.8) and correlated with the translation complexes at the corresponding location. In particular, among the cytoplasmic translation complexes, the initiation factors eIF and the termination factors eRF are very coordinated and respond very fast, while of the three elongation factors only eEF2 and eEF3 are well-coordinated, whereas the larger complex eEF1 shows a less-defined response pattern, with only the subunit eEF1-*β *clearly expressed. Overall for the class of translation complexes the pattern of activation of the response reflects closely the corresponding HL distributions [20] (eIF and eRF have short HL, eEF has not). Notice that a simple comparison of the HLs of the cytoplasmic and mitochondrial ribosomal and translation machineries (both approximately 24 min) does not show the significant difference which can be seen on the time series profiles and which is instead revealed by the phase delay analysis. For cytoplasmic ribosomal biogenesis, a similar anomaly is encountered also in the stress/stimuli responses analyzed below. For mitochondria, the same type of pattern is verified also by other complexes, for example by both the translocases located in the outer and inner mitochondrial membranes (*TOM *and *TIM*) which are known to mediate the protein import into the mitochondria, see Fig. 3.

A neat organization can be seen also in the phase of the nucleotide and amino acid metabolism: while pyrimidine and purine synthesis, as well as e.g. the CTP synthase enzyme involved in pyrimidine biosynthesis, are synchronous with the burst of transcription, the peaks for most of the enzymes involved in amino acid pathways tend to be in phase with the activation of the translational machinery. Also the synthesis of aminoacyl-tRNAs, necessary for the delivery of the amino acids to the ribosomes during translation has a similar phase. As expected, the "synthesis" pathway of an amino acid always anticipates its "degradation" pathway (see Fig. S2 in Additional file 1). In order to start translation, the initiator tRNA carrying methionine is required, and in fact, among the amino acid metabolic pathways, methionine is one of the fastest. As a matter of fact, the pathways of sulfur metabolism and of the sulfur-related amino acids (methionine, cysteine, as well as the closely related selenoamino acid metabolic pathway) present very similar and very compact time series (see Fig. S3 in Additional file 1), with an early (synchronous with the main burst) but long lasting activation (duration of the pulse is more than 100 min). This tight coordination may hint at a special role played by the sulfur pathways in the yeast population synchronization [31,32].

To conclude the protein synthesis, the nascent polypeptide chains must fold into 3D structures. The molecular chaperonin-containing T-complex and the Gim complex, which help in the folding, behave synchronously with the main burst. On the contrary, ubiquitin and proteasome, that proceed to the recognition and degradation of anomalous proteins, as well as the SCF and anaphase promoting complexes, that cause the proteolysis of the cyclin-CDK complexes, have patterns of activation which are more delayed and broadened. Actually, this class of proteolytic processes (macrocategory "folding, sorting and degradation" in Fig. 2) has the highest values of phase i.e., it has the slowest response to the transcription bursts.

The macrocategory "DNA replication and repair" (see Fig. 2) contains what remains of the "fast" responses to a large extent synchronous (protein complexes: DNA damage checkpoint, DNA repair, pre-replication, replication, replication fork, which includes all DNA polymerases, helicases and ligases, cyclin-CDK) or within a short time delay from the initial bursts of transcription. The peculiarity of this class is that the pulses are more long lived than in the "transcription" and (cytoplasmic) "translation" categories. Also the complexes regulating the cohesion and separation of sister chromatids during the S-phase (nuclear cohesion family of complexes) follow the same pattern (see Fig. S5 in Additional file 1).

Moving to the core of the cell's metabolic activity, the average phase increases further (see Fig. 2), but the main qualitative difference is on the shape of the pulses, which are now broader and often with an asymmetric rise/decay profile: still sufficiently fast activation but slower and less abrupt decay. This difference is likely to reflect the longer HL associated to these categories (all have average HL ≥ 30 min), and implies metabolic functions more overlapping than sequential. Along each metabolic pathway, the degree of correlation among enzymes catalyzing neighboring reactions is higher than it is expected (the "expected value" is inferred from a large collection of yeast microarray experiments, see Fig. S4 in Additional file 1) implying a coherent and coordinated temporal behavior along the metabolic routes. Especially for mitochondrially localized pathways such as citric acid cycle and oxidative phosphorylation the pulses are very broad, with a neat downregulation only in correspondence of the bursts of transcription and an overall profile often exhibiting a double peak on each period (occurring with a phase lag of ~100° one from the other, see Fig. S8 in Additional file 1). The four respiratory chain complexes for example follow this pattern in a fairly precise manner. As shown in Additional file 1, this double peak characteristic is often associable with pairs of genes whose products are isoenzymes oscillating in antiphase, especially for enzymes involved in oxidoreductive processes (e.g. along the pentose phosphate pathway).

### Regulation via TFs versus RBPs

**Figure 4:**
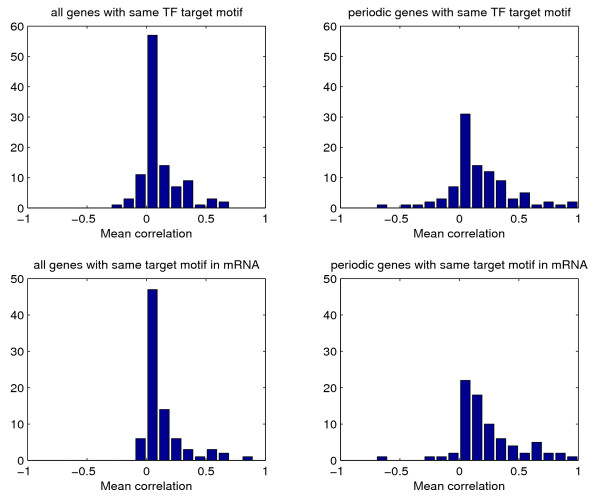
**TF regulation versus RBP regulation**. Top row: Distribution of the mean correlations for groups of genes having a common DNA motif likely to be the target of a TF [36]. Bottom row: Distribution of the mean correlations for groups of genes having a common mRNA motif likely to be the target of a RNA-binding protein (Yra1, Mex67 [34] or the five Puf proteins [23]) or having a common 3' UTR motif implicated in the stability or in the subcellular localization of the mRNA [22]. The mean correlation of a group of genes is defined as the average of the correlations between the expressions of each gene pair in the group. The mean correlations calculated for all the gene pairs are shown on the left, while on the right only the periodic genes of each group are considered.

In terms of regulatory influence, while the importance of transcription initiation via TFs is widely studied and a large amount of data (computational and experimental) is available about the binding of TFs to target genes, similar post-transcriptional systematic data on the regulation by means of RBPs are still sporadic [33]. Notable examples are mRNAs associable to the nuclear export proteins Mex67 and Yra1 [34], the Puf family of RBPs [23], and the 3' UTR motif collection of [22]. Inspired by [35], we applied these RBP lists as well as the list of TF binding sites from [36,37] to the YMC time series comparing the average correlation among genes being common targets of a TF or of a RBP. The two distributions are shown in Fig. 4. For both TFs and RBPs, only a few motifs emerge as having a significantly high correlation. The number of genes regulated by the same TF varies between 1 and 226 with a mean of 35.2, while the number of genes with a common target mRNA motif varies between 6 and 1138 with a mean of 81.7. If we draw from a null distribution representing random grouping, increasing the number of genes in a group the probability of finding a high mean correlation obviously decreases, so we expect the distribution for the second set to be tighter around 0. In our case, on the contrary, there are 6 groups out of 110 with a mean correlation ≥ 0.4 for the TF target genes (versus an expected value of 1 for random groups of genes with the same cardinalities of these groups) and 7 groups out of 83 for the genes with a target mRNA motif (versus the expected 0 for random groups with same cardinalities). This suggests that post-transcriptional regulation is more significant that transcriptional regulation in the coordination of the metabolic cycle, although the evidence is not conclusive. When checking the groups of periodic genes with high correlation we found the following significant annotations:

• 44 genes out of 56 having Fhl1p as TF and 10 genes out of 12 having Sfp1p as TF are constituents of cytoplasmic ribosomes; notice that instead other cytoplasmic ribosomal TFs such as Rap1p do not correspond to a sufficiently high correlation;

• 22 genes out of 26 having Hap4p as TF code for subunits of respiration chain complexes;

• 62 out of 220 genes whose mRNA is bound by Puf3p are annotated for mitochondrial transcription/translation (56 are part of mitochondrial ribosomes, of which 47 are periodic), see Fig. 3.

### Dynamical features of the unfolding cycle

Possible origins of the sustained oscillations are discussed at length in the literature [3,5-8,10,11,38]. Also Tu et al. explain the cycle and its time compartmentalization in terms of metabolism and redox balance [4,32,39]. Rather than adding to the list of mechanisms for metabolic regulation, by viewing each cycle as the dynamical response to a burst of transcriptional activation, this work aims at providing a characterization of the dynamics of the unfolding of the cycle, i.e., of how these "impulse responses" are progressively delayed and broadened with respect to the input pulses, and of how this correlates with the stability of the corresponding transcripts. The compactness in terms of phase and width of the early categories over repeated oscillatory cycles is an argument in favor of the existence of a single triggering event for each cycle, corresponding to the transcriptional activation bursts mentioned above. In fact, sharp, equispaced pulses are maintained in spite of the broader and less coordinated profiles of the events immediately preceding them. This hypothesis is not in contradiction with the observations about the metabolic origin of the YMC, neither with the observed alterations of the period following a genetic disruption [8,32,39] (which could in principle preserve the sequence of events described). On the contrary, it merges the metabolic control level described in [4] with an extra regulatory element which is known to play a role in dynamical contexts. In fact, the mRNA stability reflects known properties of the corresponding gene products: while mRNAs encoding transcriptional machinery or regulatory components tend to be short-lived and to turn over more quickly, transcripts encoding core enzymatic proteins are typically more stable [15,19,20]. For what is known, protein synthesis tends to follow the concentration of the corresponding mRNA [40] and to be at least as stable, if not longer-lived [41,42]. Hence, it is expected that the concentration of the gene products follows profiles that are similar to those of the mRNAs. The observation that the dynamics through a metabolic pathway can be considered as a timed and sequential process at the level of gene expression appears in several papers in the literature, see [43,44]. The same principle seems to be reflected in the YMC, although it is not observable at the level of detail investigated e.g. in [44], but more macroscopically and at genome-wide level.

### An input-output dynamical model

**Figure 5:**
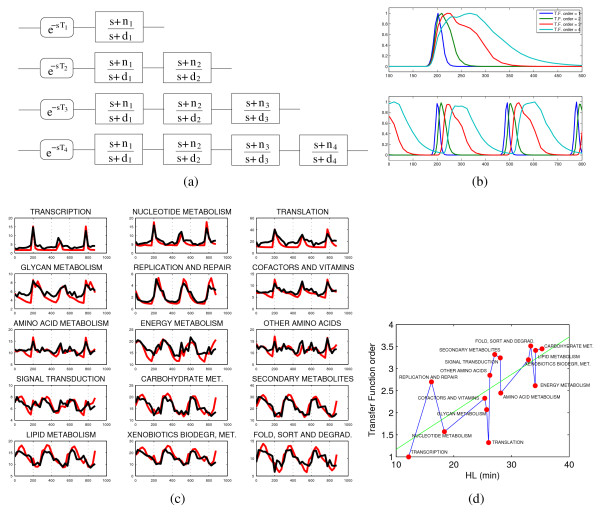
**Dynamical model of the response to a sequence of transcriptional pulses**. Dynamically, the response of the system to the sharp pulses of transcriptional activation can be modeled in terms of input-output transfer functions (i.e., convolution integrals in the Laplace domain, see Methods for details). The main feature of a simple zero-pole transfer function with low-pass characteristic is that in correspondence of an impulse-like input it yields an output which is a smoothed and broadened version of the input. Concatenations of such zero-pole transfer functions describe accurately the progressive broadening and delaying of the YMC gene expression time series. Typical time profiles obtained for transfer functions of order 1 to 4 sketched in (a) are shown in (b). The top plot in (b) shows the larger kernels obtained by concatenating up to 4 first order transfer function blocks. The lower plot in (b) shows how consecutive impulse responses look like for the various orders of transfer functions and an extra delay element as in eq. (4). A simple fitting of the *n*_*i*_, *d*_*i *_and *T*_*i *_parameters and of the best model order for each gene allows to accurately reconstruct the average profiles for the 15 macrocategories of Fig. 2 (in (c) the model-based time courses are shown in red). With the usual exception of the category "translation", the best transfer function order is roughly proportional to the corresponding HL values, coherently with the other variables discussed in the paper.

In terms of dynamical models, the progressive broadening and smoothing of the response to a sequence of (transcriptional) pulses can be described by means of simple linear input-output models (i.e., transfer functions in the Laplace domain) of increasing order having "low-pass" characteristics. As the time constant of this low-pass filter is essentially given by the HL of the mRNA, this type of model naturally predicts the correlation HL-pulse width. In order to describe correctly also the phase along the cycle, a time delay is added to the response, see Methods for a thorough description and Fig. 5(a, b) for an example. If the order of such a fitted minimal dynamical model is used to sort the annotated macrocategories of Fig. 2, we still recover both the same expected cascade of events and the same direct proportionality with HL, see Fig. 5, meaning that even in terms of the simplest possible dynamical model the kernels providing the best fitting become increasingly complex as we progress through the cycle. This is of course expected as the mRNAs gradually pass from fast turnover to high stability.

### A common dynamical gene expression program

As the YMC is obtained only in particular conditions (long-term continuous cultures in chemostats), an intriguing question is whether this highly organized unfolding of the dynamical response to pulses of transcriptional activation is peculiar only of the YMC or can be observed also in other experimental conditions. For this purpose, we consider the gene expression response of steady-state yeast to pulses of glucose described in [28]. In this case, the yeast shows a transient dynamical response but no oscillatory behavior. Furthermore, the transient peaks are more or less synchronous for all genes, i.e., there is no time-ordering in the dynamics, unlike in the YMC.

**Figure 6:**
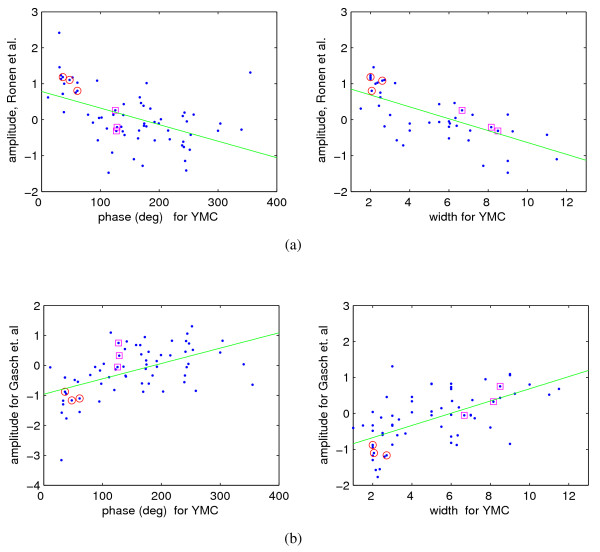
**Common unfolding of gene expression responses**. The short-term responses of steady-state yeast to pulses of nutrient discussed in [28] and the stress responses of [29] show a transient peak of up/down regulation. The peaking times are substantially uniform on the genes. For each gene we compute the maximal signed amplitude at the peak and lump together genes belonging to each of the known protein complexes (see Fig. S5 in Additional file 1). If for [28] we compare this amplitude with the phase (left) and the pulse width (right) of the corresponding genes for the YMC, we can observe that both scatter plots have a consistent anticorrelation: complexes upregulated in the glucose stimulations of [28] correspond roughly to "early" complexes in the YMC and also to genes with a fast turnover. At the other end, complexes downregulated in [28] are late in the YMC and are more stable, see (a). This shows how, in spite of different growth and stimulation conditions, the gene expression program is substantially faithful. On the contrary stressful stimuli such as those described in [29] yield correlated pattern with phase/width of the YMC (b). Just like for the YMC, for both types of responses cytoplasmic translation behaves differently from the mitochondrial one. In red circles the first 3 complexes of Fig. 3(c) are highlighted, in magenta squares their mitochondrial counterparts. Hence the anomaly represented by cluster 9 of Fig. 1 with respect to the HL classification is confirmed by these other dynamical responses.

However, if for a gene we compare the maximal signed amplitude of each expression profile on these time series with the corresponding phase and pulse width in the YMC, a sizable anticorrelation emerges, see Fig. 6(a). If, on the contrary, we consider the stress responses time series of [29], the YMC phase/pulse width turn out to be positively correlated (rather than anticorrelated) with amplitude, i.e., categories appearing early in the YMC tend to be downregulated in most stress responses, while "late phase" categories tend to be upregulated, see Fig. 6(b). It is known that in the stress responses genes annotated for ribosomal proteins and/or RNA metabolism are in general downregulated, while e.g. respiratory genes (such as those of the citric acid cycle and of the oxidative phosphorylation) become upregulated [29]. On Fig. 6, notice that also in all these responses cytoplasmic ribosomes (cluster 9 in Fig. 1) are aligned with the rest of the (cytoplasmic) transcriptional/translational machinery rather than with the assigned HL values.

The conclusion of this analysis is therefore that in intrinsically dynamical contexts some form of common response might indeed be taking place, although exerted by different means. Such genome-wide coordinated response shows a graded ordering which reflects the degree of stability of the genes involved.

## Conclusion

In [4,39] the time compartmentalization of the cycle is interpreted in terms of the need to accumulate sufficient products from the metabolic reactions in order to move on to the next phase of the cycle and to autoinduce further cycles of oscillations. This picture is not contradicted by our observations.

If, as we do in this paper, rather than looking at the YMC merely as cyclic oscillations, we study it as a highly organized dynamical response to pulses of transcriptional activation, then this response can be analyzed in much more detail at genome-wide level and we can observe how an important role in the coordination seems to be played by the mRNA turnover rate. The self-sustained character of what we consider the most upstream event of the cycle, the transcriptional activation burst, can still be conditioned to the accumulation of the required metabolites, while the unfolding of the cycle, which from the analysis of [4] is already known to be functional to the distribution of e.g. the redox load of the cells, is enriched of an extra, intrinsically dynamical feature. This feature is a fine-graded detail of our notion that genes with a fast turnover are typically regulatory, while slow genes are enzymatic and metabolic [15,19]. It can be used to describe the sequence of events occurring in the YMC as a "natural" gene expression program.

Extrapolating from the specific YMC context, the ordered pattern of events described for the YMC is to a good extent similar to that found on other intrinsically dynamical contexts such as the stress/stimuli responses. Whether the mRNA stability is the cause of this coherent behavior or is simply another effect of a more profound regulatory mechanism is a question to which we cannot provide a definitive answer at the moment.

## Methods

### Data sources

The YMC time series of [4], the compendium of 790 gene profile experiments (all performed with the Affymetrix GeneChip Yeast Genome S98 platform) and the data series from [28] were downloaded from Gene Expression Omnibus [45]. The time series of [28] are performed with cDNA, hence values of the area under the profiles are intended as relative (to the basal mRNA abundance). For each gene, the values obtained for the two different glucose stimuli are averaged. Five stress responses from [29] (two heat shocks of different amplitude, hydrogen peroxide, diamide, and sorbitol responses) are considered. The amplitudes are averaged over the five data series (the signs of these responses are known to be highly similar, see [29]).

The metabolic pathways used are those of the Kyoto Encyclopedia of Genes and Genomes (KEGG) [46]. Also the assembling into the 15 macrocategories follows the KEGG hierarchy.

The HLs are computed averaging the values of the three experimental datasets [17,18,20]. While the magnitudes of the HLs in the three collections show some differences, in "normalized" terms (looking e.g. at rank-ordered values), the agreement between the three sets is sufficiently good, see [17] for a comparison. No turnover data specific for long-term continuous cultures are currently available. However, it is not unlikely that even in this setting the relative differences of HL rates (and also their ordering) remains more or less unchanged. In any case, we expect the correlation phase/HL to improve in presence of more tailored mRNA turnover data.

### Time series analysis

To each of the genes labeled as periodic, we associated a phase, computed maximizing the correlation with respect to a train of 360 shifted sinusoids (resolution of 1°). The 0 phase was chosen so as to anticipate of ~30° the "crucial" transcription bursts [see Additional file 1]. Given that the period is approximately 287.5 minutes (see Fig. S1 in Additional file 1), the phase delay *ϕ *can be transformed into time delay *τ *by means of the relation $\tau =\phi \frac{287.5}{360}$. Under the convention for the 0 phase, each period "begins" approximately 24 min before the transcription bursts. For each gene, the pulse width is computed estimating on each period the interval in which the expression level stays above the median value across consecutive samples.

### Least squares regressions

The p.values for the least squares regressions in Fig. 1, 2 and 5 are computed via Fisher test statistics [see Additional file 1].

### A minimal dynamical model: low-pass transfer functions and their dynamical system realizations

The aim of this Section is to set up a minimal dynamical model describing the response to the periodic bursts of transcriptional activation represented as "impulsive inputs" to the system. Such a model has to be able to reproduce the following features observable in the dataset:

• impulse responses get delayed and broadened in a way which is roughly proportional to HL;

• profile changes get progressively less steep with HL;

• the system "discharges" completely (i.e. the mRNA concentrations return to a basal level) in absence of further pulses.

At the same time, to be internally consistent a dynamical model has to:

• respect causality (i.e., be non-anticipating);

• preserve positivity of the mRNA concentrations.

In the Engineering practice of Systems Theory, one of the most elementary formalism that can be used to build dynamical models is the input-output design based on Laplace transform and elementary transfer functions [47], see e.g. [48] for an application to a transcriptional time series.

The concentration of mRNA of a gene *y *can be described as the response to the pulse of transcriptional activation *u *by the linear integral

(1) $$y(t)=\int_{0}^{t}g(t-\tau )u(\tau )d\tau .$$

In the Laplace domain, a convolution integral such as (1) corresponds to

(2) $$Y(s)=ℒ\left[\int_{0}^{t}g(t-\tau )u(\tau )d\tau \right]=G(s)U(s)$$

where *s *is the Laplace variable and *G*(*s*) is called a transfer function. If *u*(*t*) is a perfect impulse *δ*_0 _(Dirac delta) then *U*(*s*) = ℒ[*δ*_0_(*t*)] = 1. When the transfer function *G*(*s*) represents a linear differential equation (i.e. it derives from a linear convolution such as (1)), it can be expressed as a rational polynomial in the Laplace variable *s*. A simple such polynomial is

(3) $$G_{1}(s)=\frac{s+n_{1}}{s+d_{1}}$$

where *s *= -*d*_1 _is called the pole of *G*_1 _and *s *= -*n*_1 _its zero. Choosing *d*_1 _> 0 the transfer function is stable (the pole is in the left half of the complex plane), i.e., a bounded input will always result in a bounded output. When *n*_1 _> 0 the system is said to be minimum phase. In this context this is an important condition in order to guarantee positivity of the output signal for all times. The requirements above can be translated into easy-to-handle design specifications on the values of the poles and zeros of the transfer function. For example, the first requirement (at least for what concerns pulse broadening) is met by the class of so-called low-pass filters, the most basic of which has the form given in (3), provided we choose 0 <*d*_1 _<*n*_1_. The term "low-pass" literally means that low frequencies in the input signal pass unchanged through the transfer function *G*_1_(*s*), while high frequencies get damped, hence the impulsive input exits from *G*_1_(*s*) smoothed and with more gentle slopes. Such a transfer function is proper and therefore respects causality; it discharges completely as required (since it has no integrator, i.e., no factors of the form 1/*s *in *G*_1_(*s*)). Strictly speaking, it is not a positive filter [49], however as long as *u*(*t*) > 0 and 0 <*d*_1 _<*n*_1 _it is also *y*(*t*) > 0. In the Laplace domain, a time delay *T*_1 _has Laplace transform equal to $e^{-T_{1}s}$. This operator does not add poles or zeros to (3) but yields the irrational transfer function

(4) $$y=G_{1}(s)e^{-T_{1}s}u.$$

In the time domain, each convolution integral (1) can be expressed as a linear input-output systems (of ordinary differential equations). For the transfer function in (3) and the delay operator in (4) this corresponds to

$$\begin{array}{c} \frac{dx(t)}{dt}=-d_{1}x(t)+(n_{1}-d_{1})u(t-T_{1})\\ y(t)=x(t)+u(t-T_{1}) \end{array}$$

i.e., the pole *d*_1 _plays the role of "degradation rate" while the activation amplitude is proportional to *n*_1 _- *d*_1 _(> 0). The typical impulse response of a low-pass filter transfer function such as (3) is shown in the top plot of Fig. 5(b). Given a pulse shape, the capabilities of a single low-pass filter in terms of broadening and smoothing of the responses are limited, hence, in order to obtain a progressive effect of delayed and broadened impulse responses, several delayed low-pass filters should be put in cascade. For example the order-2 transfer function obtained concatenating 2 filters is

$$G_{2}(s)=\frac{\left(s+n_{1})(s+n_{2}\right)}{\left(s+d_{1})(s+d_{2}\right)},$$

or, in the time domain,

$$\begin{array}{c} \frac{dx_{1}(t)}{dt}=-d_{1}x_{1}(t)+(n_{1}-d_{1})u(t-T_{2})\\ \frac{dx_{2}(t)}{dt}=-d_{2}x_{2}(t)+(n_{2}-d_{2})(x_{1}(t)+u(t-T_{2}))\\ y_{2}(t)=x_{1}(t)+x_{2}(t)+u(t-T_{2}). \end{array}$$

In this case both *d*_1 _and *d*_2 _contribute to forming the degradation profile of the mRNA concentration *y*_2_(*t*). Likewise both dynamical variables *x*_1 _and *x*_2 _contribute to shape the pulse of a gene. Typically this model induces a steeper upregulation and a slower degradation front, coherently with what we observe on the YMC time series. The intermediate variables *x*_*i *_are only meant to describe the complexity of the input-output relationship. Qualitatively, they might reflect intermediate steps in the gene expression program. For example, the transcription of the genes of the central metabolism is activated downstream of the genes for translation and amino acid synthesis, which in their turn follow the principal bursts of transcription machinery (polymerases and other RNA processing components). Downstream activation of the genes of a category translates in this modeling framework into delayed and broadened pulses. Typical output responses for 1, 2, 3, and 4 such concatenated blocks are shown in Fig. 5(b).

A simple parameter search can be set up to identify values of *n*_*i*_, *d*_*i *_and *T*_*i*_, *i *= 1,...,4, that guarantee for each gene a sufficiently well-reproduced time course. The best transfer function order for each gene is identified as that maximizing the correlation between true and model-based time series.

### HL and the short-period YMC of [3]

The HL of a gene is defined as the time needed to halve the concentration of mRNA in absence of new transcription. Hence in order for a "full" degradation of mRNA to be observed, the interval between two consecutive waves of transcription has to be at least twice or three times the HL. For yeast, the mean HL extrapolated from [17,18,20] is ~26 ± 17 min. Hence for the long-period YMC the response to bursts of transcription has the time to exhaust completely before the arrival of the next wavefront. On the contrary, for the short-period YMC described in [3] the period is approximately 40 min, meaning that excitation and degradation fronts are substantially overlapping.

## Abbreviations

HL: half life; KEGG: Kyoto Encyclopedia of Genes and Genomes; RBP: RNA-binding protein; TF: transcription factor; YMC: yeast metabolic cycle.

## Authors' contributions

NS and CA conceived the study, analyzed the data and drafted the manuscript. MZ contributed to the data analysis and the interpretation of the results. LF contributed to the interpretation of the results. All authors read and approved the final manuscript.

## Supplementary Material

Additional file 1 **Supplementary notes.** Supplementary method information, results and further comments. Click here for file
