# Supplementary material for: mRNA stability and the unfolding of gene expression in the long-period yeast metabolic cycle
Source: BMC Syst Biol. 2009 Feb 6;3:18. doi: 10.1186/1752-0509-3-18 (PMC2677395; doi:10.1186/1752-0509-3-18)
Supplement: Additional file 1 — Supplementary notes. Supplementary method information, results and further comments. [file 1752-0509-3-18-S1.pdf]

SUPPLEMENTARY NOTES
for
mRNA stability and the unfolding of gene expression in the long-period yeast metabolic cycle

N. Soranzo[†], M. Zampieri[†], L. Farina[‡] and C. Altafini[†]

† SISSA, via Beirut 4, 34014 Trieste, Italy
‡ Università degli Studi di Roma “La Sapienza”, via Ariosto 25, 00185 Roma, Italy

January 13, 2009

Contents

1 Methods **2**
- 1.1 Other data sources 2
- 1.2 Time series analysis 2
- 1.3 Least squares regressions 2
- 1.4 Clusterization 3

2 Other associations among functional categories **4**
- 2.1 More compartmentalized categories 4
- 2.2 Signaling proteins 4
- 2.3 Weakly periodic categories 4

3 Double peak and anticorrelated isoenzymes. **4**

4 Central metabolism **5**
- 4.1 Periodic pattern analysis in the central metabolism 5
- 4.2 Glucose-regulated carbon metabolism 6

5 Further comments **6**
- 5.1 Direct versus clustered correspondences phase/HL 6
- 5.2 Synchronization and pulse width 6

List of Figures

- S1 Computation of the period of the bursts in the YMC 8
- S2 Phase, HL, pulse width and correlation along the KEGG pathways 9
- S3 Correlation-based clusterization of the KEGG pathways 10

S4 Correlation between genes along the KEGG metabolic pathways 11
S5 Phase, HL, pulse width and correlation for a few protein complexes 12
S6 Anticorrelated isoenzymes 13
S7 Phase of the periodic genes on the central metabolism 14
S8 Time course of the central metabolic pathways 15
S9 Time course of some anticorrelated isoenzymes 15
S10 Enzymatic genes for ATP/ADP/AMP and O_2 reactions 16

1 Methods

1.1 Other data sources

The protein complexes were downloaded from the MPACT subsection (http://mips.gsf.de/genre/proj/mpact/) of the CYGD database at MIPS. Only complexes manually annotated from the literature are considered; those obtained from high throughput experiments are disregarded (according to the MIPS classification scheme these last are labeled "550").

1.2 Time series analysis

As in [1], genes are filtered using a periodogram test. In order to retain only genes with a well-defined periodicity, we fixed a more selective p.value than [1] thereby reducing the number of genes to 1951.

The period of the YMC, computed in the time domain looking at the most impulsive-like categories (in Fig. S1 the 3 RNA polymerases), is estimated as 287.5 min (see Fig. S1 for a detailed description).

To each of the 1951 genes labeled as periodic, we associated a phase, computed maximizing the correlation with respect to a train of 360 shifted sinusoids (resolution of 1°). Means and standard deviations of the phases of periodic signals must be computed "mod 360°", and are normally subject to large numerical errors and ill-conditioning. A typical example is the following: assume two periodic genes are assigned the phases $\phi_1 = 350^\circ$ and $\phi_2 = 6^\circ$. Owing to the 360° periodicity, the peaks of the two genes are very close, but the average phase is $(\phi_1 + \phi_2)/2 = 178^\circ$, which is obviously wrong. The correct answer requires a shift from the principal values of the periodic signal: $(\phi_1 - 360^\circ + \phi_2)/2 = -8^\circ$. To avoid problems with biased mean values and/or the appearance of inelegant negative phases around the "crucial" transcription bursts, the 0 phase was chosen so as to anticipate of $\sim 30^\circ$ these events.

Given that the period is approximately 287.5 minutes, the phase delay ϕ can be transformed into time delay τ by means of the relation $\tau = \phi \frac{287.5}{360}$. Under the convention for the 0 phase, each period "begins" approximately 24 min before the transcriptional bursts.

For each gene, the pulse width is computed estimating on each period the interval in which the expression levels stays above the median value across consecutive samples.

1.3 Least squares regressions

In Fig. 1 of the paper, the least square fitting in the HL/phase plot is given by the equation

$$\phi = 9.25 \text{ HL} - 104.8^\circ, \quad R^2 = 0.86, \quad \text{p.value} \sim 10^{-7}.$$

The corresponding p.value is computed via a Fisher test statistics. Since we have determined the period as 287.5 min and the zero phase $\sim 30^\circ$ before the impulsive bursts shown for example in Fig. S1, the equation in terms of time delay with respect to the bursts, $\tau_t \simeq 0.8(\phi - 30^\circ)$, is

$$\tau_t \simeq 7.38 \text{ HL} - 128.8 \text{ (min)}.$$

Within most clusters, the standard deviation in terms of ϕ is minimal; it is higher in terms of HL, see Table 1 of the paper. Hence if we use weighted least squares regression, while the fitted curves we get are still very similar in the range of values of interest, the differences are in the coefficient of determination R^2:

method	regression	R^2	p.value
l. s. weighted w.r.t. ϕ	$\phi = 8.95$ HL $- 101.24$	0.92	$\sim 10^{-9}$
l. s. weighted w.r.t. HL	$\phi = 9.03$ HL $- 99.76$	0.54	$\sim 10^{-4}$

If ω = width of the pulses, then the corresponding least squares fits are

method	regression	R^2	p.value
l. s.	$\omega = 0.24$ HL $- 1.05$	0.72	$\sim 10^{-5}$
l. s. weighted w.r.t. ω	$\omega = 0.21$ HL $- 0.99$	0.32	$\sim 10^{-5}$
l. s. weighted w.r.t. HL	$\omega = 0.23$ HL $- 0.71$	0.31	$\sim 10^{-2}$

Repeating the linear regression for the three plots in Fig. 2(b) of the paper we get:

- phase/HL (top plot)

method	regression	R^2	p.value
l. s.	$\phi = 6.84$ HL $- 33.05$	0.64	$\sim 10^{-4}$
l. s. weighted w.r.t. ϕ	$\phi = 4.39$ HL $+ 27$	0.82	$\sim 10^{-6}$
l. s. weighted w.r.t. HL	$\phi = 5.26$ HL $+ 6$	0.68	$\sim 10^{-4}$

- width/HL (middle plot)

method	regression	R^2	p.value
l. s.	$\omega = 0.15$ HL $+ 1.41$	0.44	$\sim 10^{-3}$
l. s. weighted w.r.t. ω	$\omega = 0.19$ HL $+ 0.5$	0.53	$\sim 10^{-3}$
l. s. weighted w.r.t. HL	$\omega = 0.11$ HL $+ 2.58$	0.8	$\sim 10^{-5}$

- width/phase (bottom plot)

method	regression	R^2	p.value
l. s.	$\omega = 0.02\phi + 1.9$	0.8	$\sim 10^{-6}$
l. s. weighted w.r.t. ϕ	$\omega = 0.03\phi + 1.66$	0.96	$\sim 10^{-10}$
l. s. weighted w.r.t. ω	$\omega = 0.03\phi + 1.12$	0.8	$\sim 10^{-6}$

Finally, for the dynamical model of Fig. 5 of the paper, if ψ is the order of the transfer function model used, $\psi \in [1,\ 4]$, we have

method	regression	R^2	p.value
l. s.	$\psi = 0.09$ HL $+ 0.32$	0.52	$\sim 10^{-3}$
l. s. weighted w.r.t. HL	$\psi = 0.06$ HL $+ 1.1$	0.73	$\sim 10^{-5}$

1.4 Clusterization

The clusterization of the time profiles in Fig.1 of the paper is performed via a k-means algorithm using as distance a nonnormalized correlation function. Varying the number of clusters and/or the (randomly chosen) initial cluster assignments, the results (in terms of the regressions) are basically unchanged. A k-means algorithm is also used to cluster the KEGG pathways in Fig. S3. In this case as distance we use the average of the pairwise correlations between all genes of every two pathways.

2 Other associations among functional categories

2.1 More compartmentalized categories

Other categories which can be associated with a particular cellular compartment emerge from the joint analysis of pathways and protein complexes. For example lipid biosynthesis, which is to a large extent localizable in the endoplasmic reticulum (ER) has a phase comparable to the translocon family of complexes (Fig. S5) which is composed of the Sec61 protein translocator, the signal recognition particle which binds the ER-specific sequence on the nascent polypeptide chain and the signal peptidase that cleaves it off.

2.2 Signaling proteins

Complexes with eminently intracellular signaling functions, such as the antagonistic cAMP-dependent protein kinase and serine/threonine phosphoprotein phosphatases (respectively phosphorylator and dephosphorylator of signaling proteins) have similar patterns of expression, similar timing during the YMC and high Pearson correlation (at least for what concerns periodic genes).

2.3 Weakly periodic categories

Several categories linked to transcriptional activation or RNA processing, like the histone acetyltransferase enzyme or the nuclear processing complex family (3'-end pre-RNA processing factors CFI and II and 3'-end polyadenylation factors PFI) or the chromatin assembly complex, seem to be evading the tight phase coordination. However, this is mostly due to the evanescent periodic pattern, if any, of the corresponding genes. Likewise for the nuclear pore complex, which assists the export of mature mRNA through the nuclear envelope: most of its genes in fact show bursts which are synchronous with the initial pulses but of very small amplitude, thus ambiguous in terms of temporal classification.

3 Double peak and anticorrelated isoenzymes.

Especially for mitochondrially localized pathways such as citric acid cycle and oxidative phosphorylation the pulses are very broad, with a neat downregulation only in correspondence of the bursts of transcription and an overall profile often exhibiting a double peak on each period (occurring with a phase lag of $\sim 100^\circ$ one from the other, see Fig. S8). The four respiratory chain complexes for example follow this pattern in a fairly precise manner.

In order to investigate the meaning of this double peak characteristic, we consider genes whose products are classified as isoenzymes. If we look at the correlation for all pairs of isoenzymes, see Fig. S6(a), we see that restricting to periodic genes an almost bimodal distribution emerges, with a significant percentage (43 out of 210) of isoenzyme pairs being anticorrelated ($R < -0.3$). This behavior has no counterpart on the distribution of expected values (computed as above by means of a large collection of microarrays). In more than 50% of these anticorrelated pairs the pattern of activation in the time course is similar (see Fig. S6(c)), with one of the two isoenzymes exhibiting a deep and prolonged downregulation immediately following the transcription bursts. The majority of these pairs is involved in oxidoreductive processes, like, for example, *SDH1*-YJL045W, *SDH3*-YMR118C, *SDH4*-YLR164W (all subunits of succinate dehydrogenase), or the NADP-dependent isocitrate dehydrogenase pairs *IDH1-IDP3*, *IDH2-IDP3*, *IDP1-IDP3*, or the plasma membrane H+-ATPase isoenzymes *PMA1-PMA2*, or the NADH dehydrogenase pairs *NDE1-NDE2*. Three among the most anticorrelated pairs of isoenzymes showing this pattern are located along the pentose phosphate pathway, two on the cytosolic oxidative branch (*SOL3-SOL4* and *GND1-GND2*), the third (the transketolases *TKL1-TKL2*) downstream. The pentose phosphate pathway synthesizes NADPH, which is the major source of reducing equivalents and, according to [2, 3], plays a major role in the establishment of the cycle. Also the most anticorrelated isoenzymes in the glycolysis pathway, the alcohol dehydrogenases, have a similar pattern: *ADH1* and *ADH3* (reducing acetaldehyde to ethanol) versus *ADH2*

(catalyzing the reverse reaction), see section 4 for a more detailed analysis of the periodic pattern in the central metabolism.

4 Central metabolism

4.1 Periodic pattern analysis in the central metabolism

From Fig. S8, it seems that the long bursts of the citric acid cycle and oxidative phosphorylation genes could be composed of two distinct adjacent phases for each period. Similarly, the profiles of the anticorrelated isoenzyme pairs mentioned in section 3 and reproduced in Fig. S9, show that of the two recurrent patterns described in Fig. S6(c), one resembles the mitochondrial transcription/translation burst (upregulation approximately in the interval 225÷375 min interval and periodically thereafter), the other is more delayed (interval 300÷450 min) and characterized by a deep downregulation during and after the main transcription bursts (200÷275 min). The alcohol dehydrogenases isoenzymes are "prototypes" of the 2 patterns: *ADH1* and *ADH3* (respectively cytosolic and mitochondrial, both reducing acetaldehyde to ethanol) follow the first, while *ADH2* (using ethanol as substrate) follows the second.

The first pattern (*ADH1*/*ADH3*) is synchronous with the hexokinases catalyzing the initial glucose phosphorylation: of the three isoenzymes, *HXK2* has the earliest response but is also more rapidly repressed, while *HXK1* is more long-lived and is expressed, together with the glucokinase *GLK1*, also in the other interval [4]. Quite unexpectedly, the enzyme for the final irreversible step of glycolysis, pyruvate kinase (*CDC19*, as the isoenzyme *PYK2* remains constantly basal), is neither synchronous with the *ADH1*/*ADH3* and *HXK2* profile, nor with the other one (*ADH2* and *GLK1*), but rather delayed with respect to both modes (in Fig. S5 pyruvate kinase has the highest phase delay). The high level of expression of alcohol dehydrogenase in all metabolic modes suggests that pyruvate production may not be the rate-limiting step of the pathway, and that a delayed pyruvate kinase action may help meeting cellular ATP demand by distributing uniformly ATP production along the cycle (see Fig. S10). As a matter of fact, *CDC19* peaks always precede the transcription bursts (in correspondence of the downregulation of the mitochondrial genes) and fall right after that. Most of the enzymes for the intermediate steps of glycolysis do not show any significant periodic trend (see Fig. S7 for an overall view of the phase of the genes on the central metabolic pathways), although on the other irreversible reaction, phosphofructokinase (both genes *PFK1* and *PFK2*) has some degree of resemblance with *CDC19*. On the contrary, the gluconeogenesis enzymes pyruvate carboxylase (*PYC1*) and phosphoenolpyruvate carboxykinase (*PCK1*) show a strong correlation with the genes *ADH1*/*ADH3* and *HXK2*.

The acetaldehyde-ethanol exchange is part of the so-called "PDH bypass" (i.e. route alternative to the pyruvate dehydrogenase complex) for acetyl-CoA production, see [5]. The supply to this pathway (through *PDC5*) is almost continuous (except in the "valleys" of the pyruvate kinase) and the rest of the pathway, aldehyde dehydrogenase (mostly isoenzyme *ALD6*, mitochondrial) and acetyl-CoA synthase (*ACS1*, cytosolic) coordinated with *ADH2*. On the contrary, the pyruvate carboxylase branch follows *ADH1*/*ADH3*, while the direct route pyruvate/acetyl-CoA (PDH complex) is unclear (more synchronous with *ADH1*/*ADH3* though).

With the exclusion of the succinyl-CoA ligase (both *LSC1* and *LSC2*) all the steps of the citric acid cycle are more in agreement with the *ADH1*/*ADH3* pattern and are rigorously shut down during the transcription bursts. From Fig. S8, it seems that the long bursts of the oxidative phosphorylation genes overlap with both patterns. Looking at the trace of observed dO_2 (data taken from [1] and reproduced in Fig. S10(b)), citric acid cycle and oxidative phosphorylation activation seem to correspond to the maximum drop in dO_2 concentration (200÷300 min interval following the transcription burst), but they seem to persist also long after the recovery of dO_2. It must be noticed that the trace of dO_2 resembles closely the expression profile of the catalase enzymes that produce O_2 detoxifying reactive oxygen species.

4.2 Glucose-regulated carbon metabolism

There is a consistent literature on the influence of glucose abundance on gene expression [6, 7, 8, 9, 10]. On the YMC, the standard glucose activated and/or repressed signaling pathways are not expressed. For example the Snf1 serine/threonine protein kinase complex subunits *SNF1*, *SNF4*, *SIP1*, *SIP2*, *GAL83*, as well as the other regulated genes on the same pathway *MIG1*, *CAT8* and *ADR1*, do not show any significant pattern.

5 Further comments

5.1 Direct versus clustered correspondences phase/HL

Unlike Fig. 1(b) of the paper, a plot HL/phase for all periodic genes looks very scattered (see Table 1 of the paper for the HL standard deviation on each cluster). One of the reasons may be that the HL measures are widely imprecise (see comparison between HL datasets in [11]), another that HL values should probably be considered as context-specific, parametrically dependent on a set of physiological conditions and/or on trans-acting degradation factors [12, 11], while the HL values available from [11, 13, 14] represent more a "built-in" turnover rate. Nonetheless, as we lump together genes according to either expression profiles (unsupervised approach) or functional annotation (supervised approach) the linear relationship neatly emerges. It is tempting to speculate that the process of grouping genes reduces the uncertainty of the turnover rate measures and that functionally similar genes might share a similar fate (e.g. being accumulated in specific P-bodies [15]) regardless of the exact value of the turnover rate.

5.2 Synchronization and pulse width

In [1] it is affirmed that broad profiles (like those associated here to "late" categories) may be due to loss of synchronization in the population of yeast cells as they progress through the cycle(s). Based on what we show in this paper, such an interpretation is problematic: loss of synchronization during a cycle would jeopardize the entire transcriptional program on the following cycles, while on the contrary, we still see thin and precisely coordinated pulses in the fast categories. Population synchronization is often said to be influenced by rapidly diffusing molecules such as H_2S [16]. We notice here that the sulfate-related pathways are expressed only in the first part of each cycle. In spite of the high coordination and early phase, the peaks for these categories last in average 100 min, see Fig. S4.

References

[1] Tu BP, Kudlicki A, Rowicka M, McKnight SL: **Logic of the Yeast Metabolic Cycle: Temporal Compartmentalization of Cellular Processes**. *Science* 2005, **310**(5751):1152–1158.

[2] Tu BP, Mohler RE, Liu JC, Dombek KM, Young ET, Synovec RE, McKnight SL: **Cyclic changes in metabolic state during the life of a yeast cell**. *Proc. Natl. Acad. Sci. U.S.A.* 2007, **104**(43):16886–16891.

[3] Xu Z, Tsurugi K: **A potential mechanism of energy-metabolism oscillation in an aerobic chemostat culture of the yeast *Saccharomyces cerevisiae***. *FEBS J.* 2006, **273**(8):1696–1709.

[4] Rodríguez A, de la Cera T, Herrero P, Moreno F: **The hexokinase 2 protein regulates the expression of the *GLK1*, *HXK1* and *HXK2* genes of *Saccharomyces cerevisiae***. *Biochem. J.* 2001, **355**(3):625–631.

[5] Rodrigues F, Ludovico P, Leão C: **Sugar Metabolism in Yeasts: an Overview of Aerobic and Anaerobic Glucose Catabolism**. In *Biodiversity and Ecophysiology of Yeasts,* The Yeast Handbook. Edited by Péter G, Rosa C, Berlin Heidelberg: Springer 2006:101–121.

[6] Cereghino GP, Scheffler IE: **Genetic analysis of glucose regulation in *Saccharomyces cerevisiae*: control of transcription versus mRNA turnover**. *EMBO J.* 1996, **15**(2):363–374.

[7] DeRisi JL, Iyer VR, Brown PO: **Exploring the Metabolic and Genetic Control of Gene Expression on a Genomic Scale**. *Science* 1997, **278**(5338):680–686.

[8] Kim JH, Brachet V, Moriya H, Johnston M: **Integration of Transcriptional and Posttranslational Regulation in a Glucose Signal Transduction Pathway in *Saccharomyces cerevisiae***. *Eukaryotic Cell* 2006, **5**:167–173.

[9] Rolland F, Winderickx J, Thevelein JM: **Glucose-sensing and -signalling mechanisms in yeast**. *FEMS Yeast Res.* 2002, **2**(2):183–201.

[10] Yin Z, Wilson S, Hauser NC, Tournu H, Hoheisel JD, Brown AJP: **Glucose triggers different global responses in yeast, depending on the strength of the signal, and transiently stabilizes ribosomal protein mRNAs**. *Mol. Microbiol.* 2003, **48**(3):713–724.

[11] Grigull J, Mnaimneh S, Pootoolal J, Robinson MD, Hughes TR: **Genome-Wide Analysis of mRNA Stability Using Transcription Inhibitors and Microarrays Reveals Posttranscriptional Control of Ribosome Biogenesis Factors**. *Mol. Cell. Biol.* 2004, **24**(12):5534–5547.

[12] Foat BC, Houshmandi SS, Olivas WM, Bussemaker HJ: **Profiling condition-specific, genome-wide regulation of mRNA stability in yeast**. *Proc. Natl. Acad. Sci. U.S.A.* 2005, **102**(49):17675–17680.

[13] Kuai L, Das B, Sherman F: **A nuclear degradation pathway controls the abundance of normal mRNAs in *Saccharomyces cerevisiae***. *Proc. Natl. Acad. Sci. U.S.A.* 2005, **102**(39):13962–13967.

[14] Wang Y, Liu CL, Storey JD, Tibshirani RJ, Herschlag D, Brown PO: **Precision and functional specificity in mRNA decay**. *Proc. Natl. Acad. Sci. U.S.A.* 2002, **99**(9):5860–5865.

[15] Sheth U, Parker R: **Decapping and Decay of Messenger RNA Occur in Cytoplasmic Processing Bodies**. *Science* 2003, **300**(5620):805–808.

[16] Sohn HY, Murray DB, Kuriyama H: **Ultradian oscillation of *Saccharomyces cerevisiae* during aerobic continuous culture: hydrogen sulphide mediates population synchrony**. *Yeast* 2000, **16**(13):1185–1190.

[17] Förster J, Famili I, Fu P, Palsson BØ, Nielsen J: **Genome-scale Reconstruction of the *Saccharomyces cerevisiae* Metabolic Network**. *Genome Res.* 2003, **13**(2):244–253.

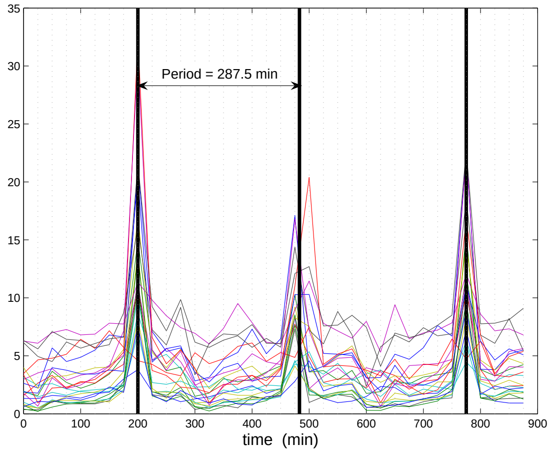

Figure S1: **Computation of the period of the bursts in the YMC**. When the period is computed via Fourier analysis, as is done in [1], the answer is 300 min. However, a closer look at the genes having impulse-like behavior (in this Figure the three RNA polymerases) reveals that the sampling is not perfectly synchronized with the period observed: in a time window twice the period (200÷775 min) there are 23 samples instead of the expected 24, and the "11.5" samples per period ratio seems to yield a more accurate matching of the peaks. The resulting period is therefore $11.5 \cdot 25 = (775 - 200)/2 = 287.5$ min. Notice how this explains why the second peak is less resolved that the first and third one in basically all Figures shown in this paper.

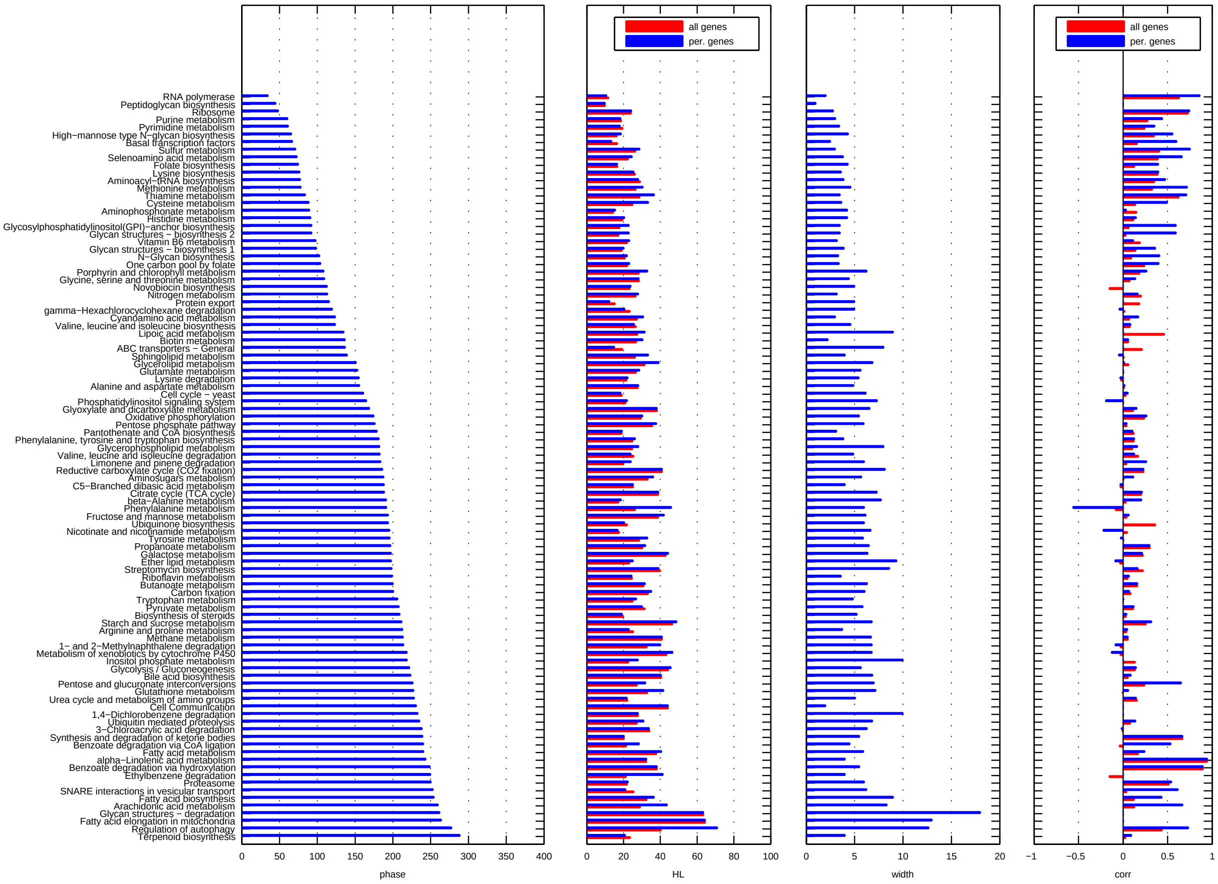

Figure S2: Average of phase, HL, area and Pearson correlation along KEGG pathways, for all genes (red) and for periodic genes (blue), sorted by phase of the periodic genes.

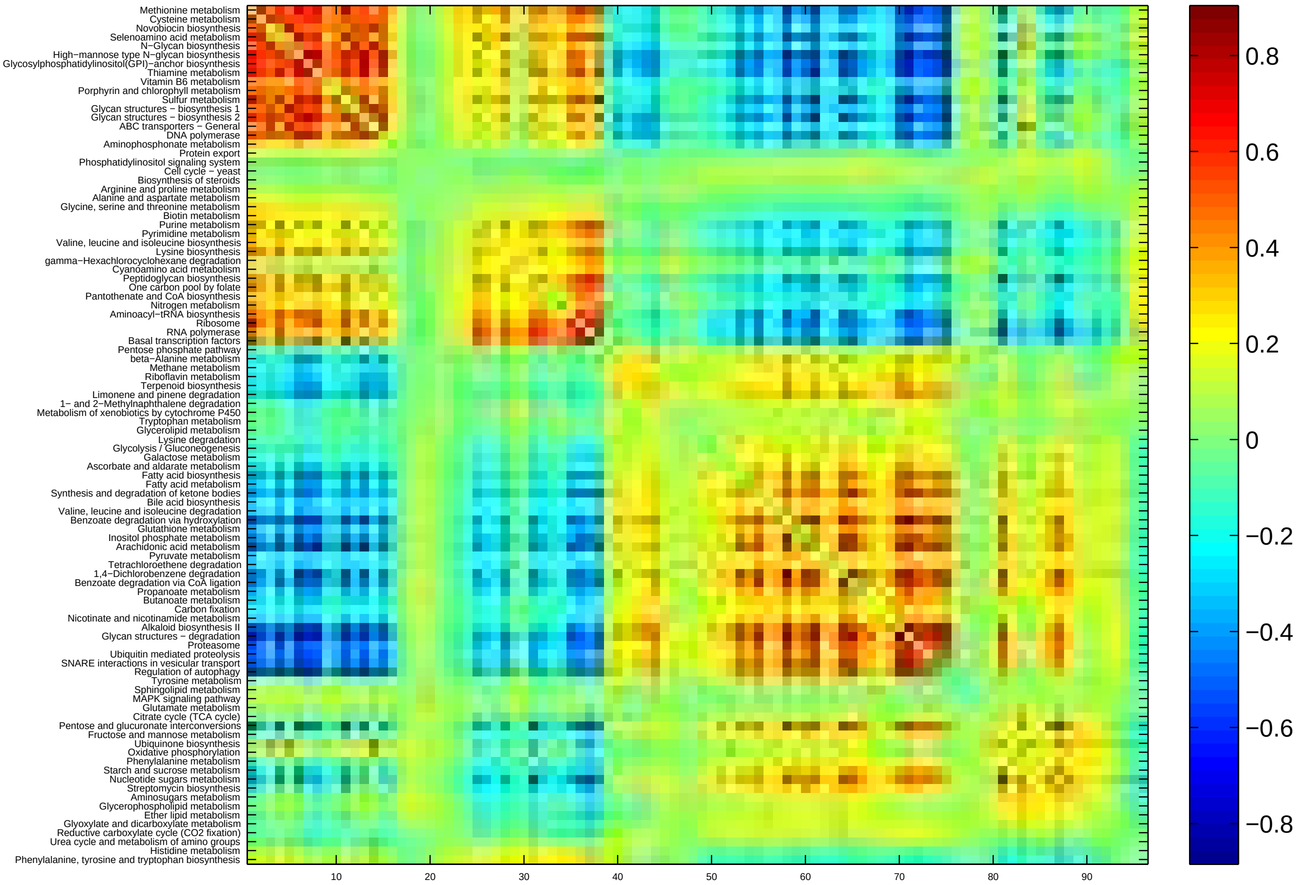

Figure S3: KEGG pathways clustered according to a k-means algorithm using as distance the average of the pairwise expression correlation between genes belonging to the pathways.

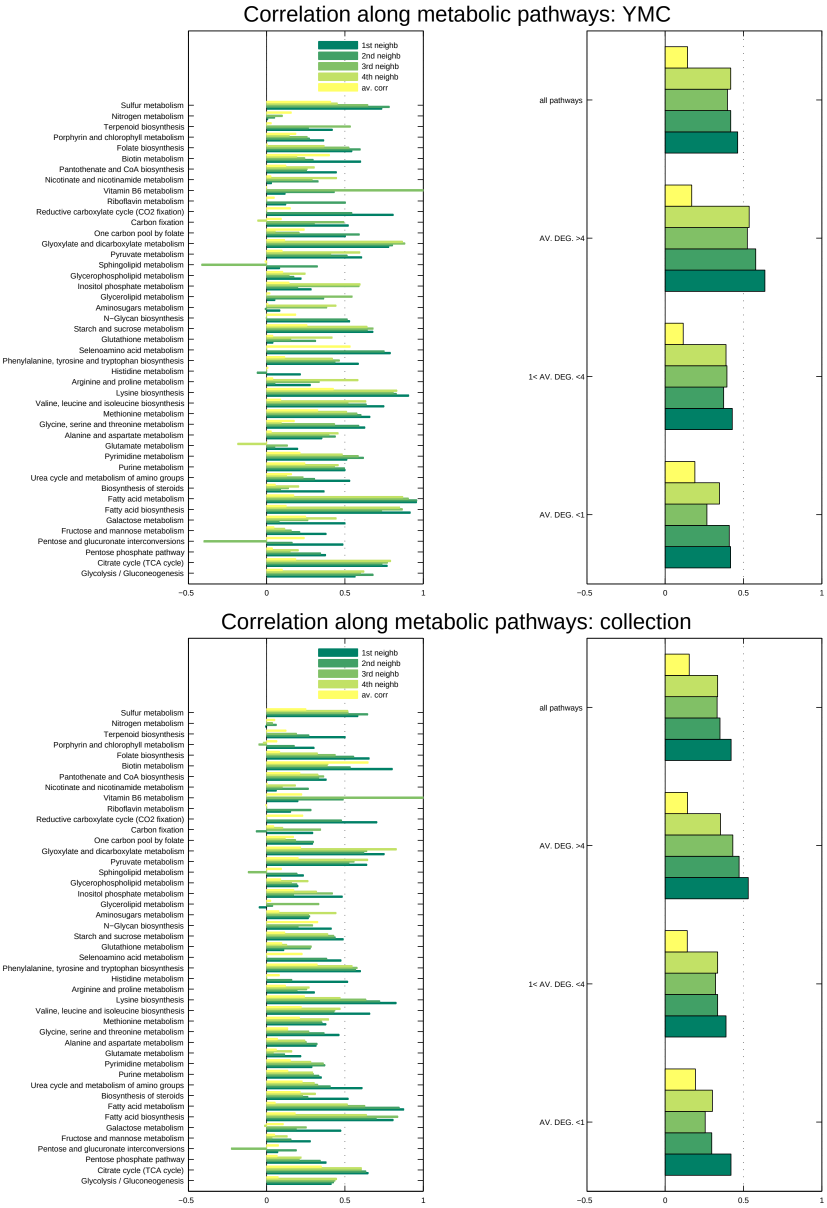

Figure S4: Correlation between genes along the KEGG metabolic pathways (genetic information categories such as transcription, translation, and DNA replication and repair are not included), computed for the yeast metabolic cycle time series (top plot) and for a collection of 790 yeast experiments (bottom plot). The correlation is computed between enzymes that are neighbors in terms of metabolic reactions: from adjacent genes to genes separated by three intermediate reactions (green scales). Averages between all genes involved in a pathway is also shown in yellow. On the right panel of both figures average values of correlations along the pathways are shown (top part of the right plots); in the remaining three plots the pathways are further grouped by average connectivity degree. Correlations are higher for more tightly connected pathways than for those having a low connectivity degree. Comparing the right hand sides of the two Figures, correlation among neighboring genes for the YMC is higher than for the collection, thus confirming the high level of functional coordination induced by the YMC along the metabolic pathways.

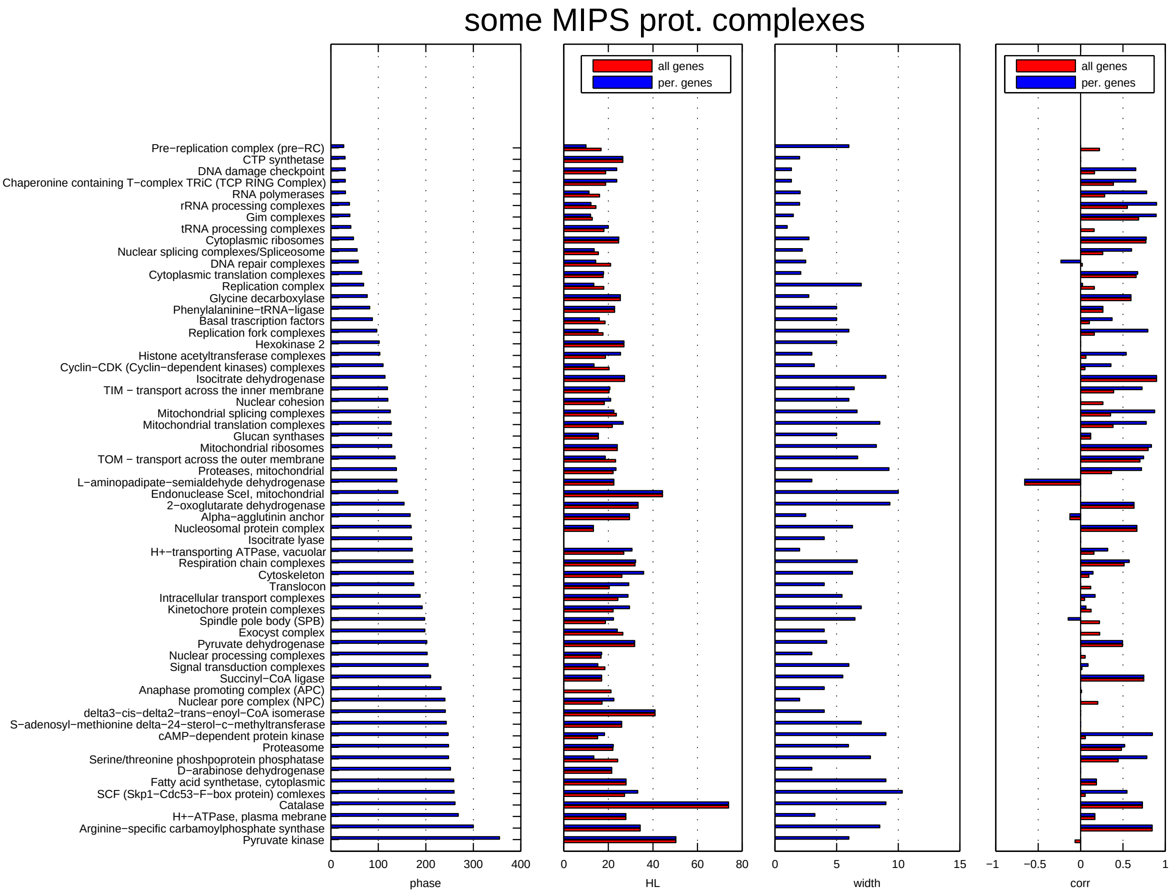

Figure S5: Average phase, HL, pulse width and intracomplex Pearson correlation for a few MIPS protein complexes, sorted by phase (on the periodic genes).

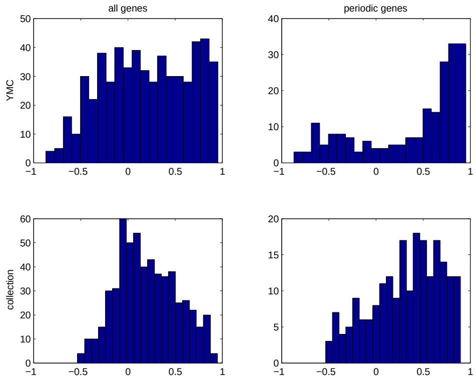

(a) Correlation between isoenzymes

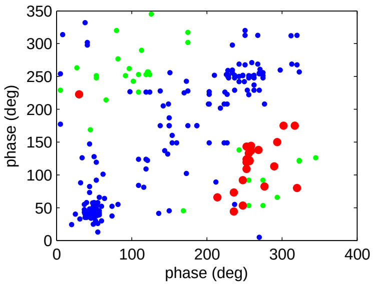

(b) Scatter plot of the phase of the periodic isoenzymatic pairs (the pairs in blue have $R > -0.3$)

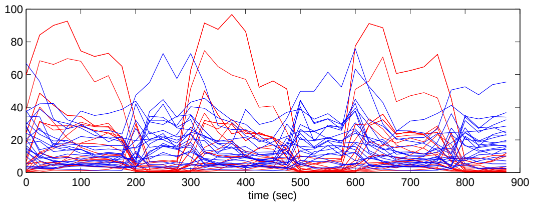

(c) Most anticorrelated isoenzymes

Figure S6: **Anticorrelated isoenzymes** for the YMC (top row in (a)) and for a collection of 790 yeast gene profiling experiments (bottom row (in (a)). The correlations between all pairs of isoenzymes in the two sets are shown on the left, while on the right only periodic pairs of genes are considered. For them in the YMC the distribution of correlations tend to a bimodal distribution, i.e., a significant subset of isoenzymes is anticorrelated and oscillates with opposite phases. The same type of anticorrelation is not visible on the reference collection. The time series of the pairs in red in the scatter plot of the phases (b) are depicted in (c). One of the two genes of these pairs (in red) is characterized by a deep valley following the transcription bursts. Most of these pairs are involved in redox processes (see Fig. S9 for more detailed plots).

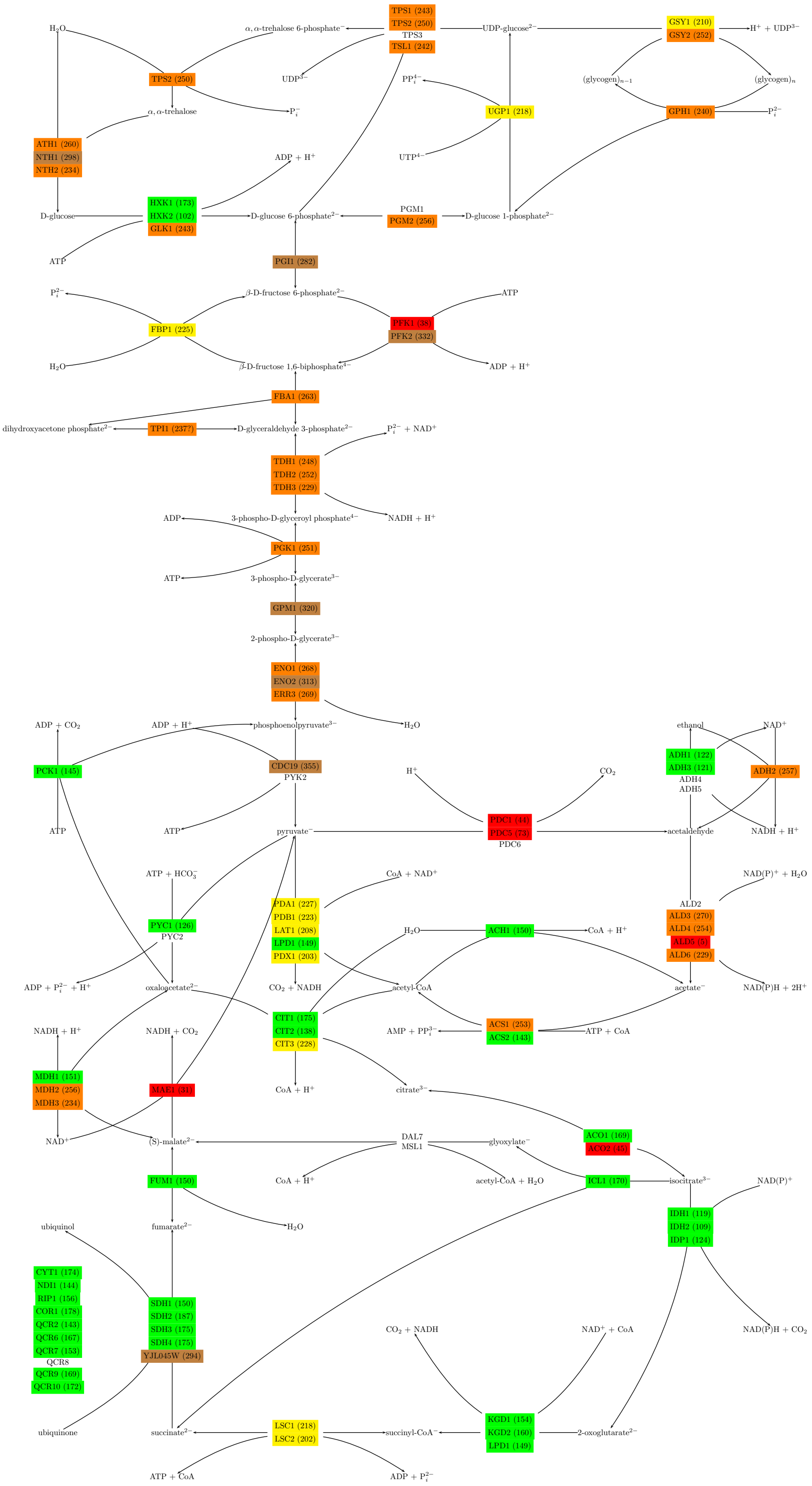

Figure S7: **Phase of the periodic genes on the central metabolism.** The color code (only for periodic genes) indicates the phase interval. Red: $0 \div 100$; green: $100 \div 200$; yellow: $200 \div 250$; orange: $250 \div 300$; brown: $300 \div 360$.

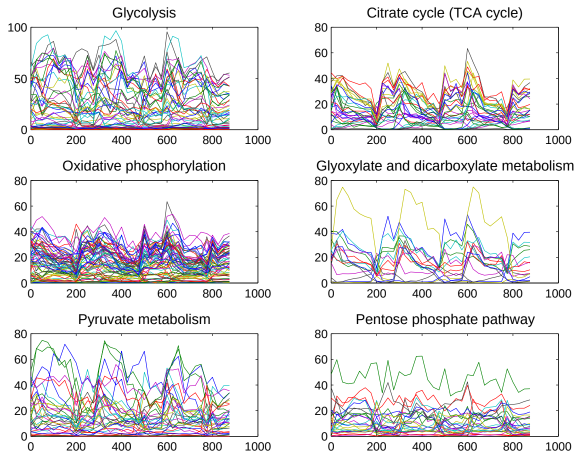

Figure S8: **Time course of the central metabolic pathways**.

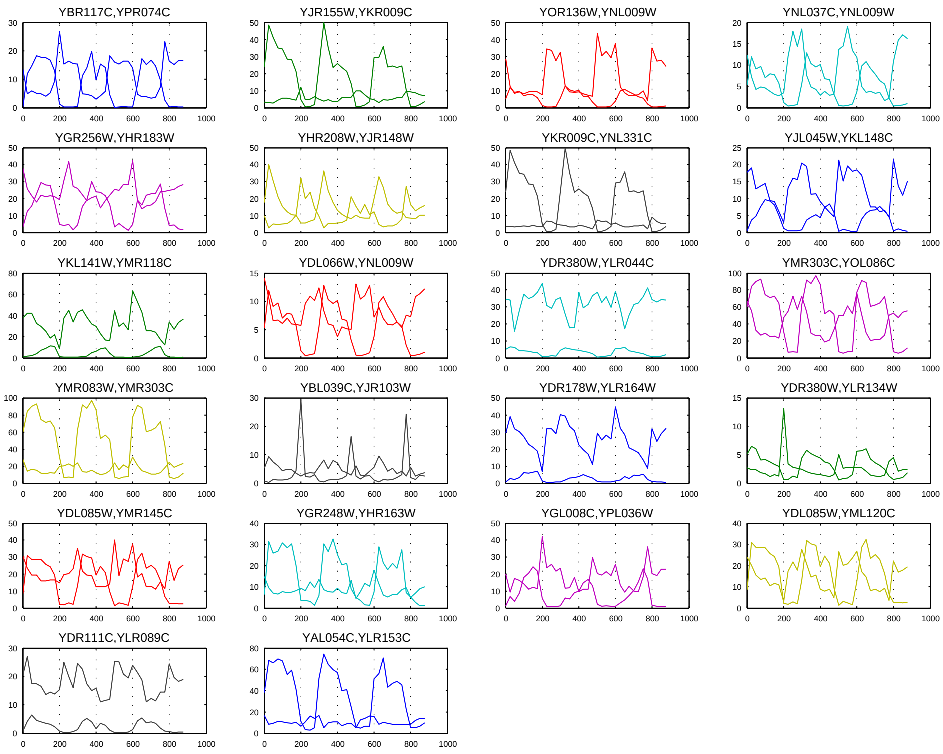

Figure S9: **Time course of some anticorrelated isoenzymes**. The pairs of genes shown are those depicted in Fig. S6(c), characterized by periodic “valleys” right after the transcription bursts.

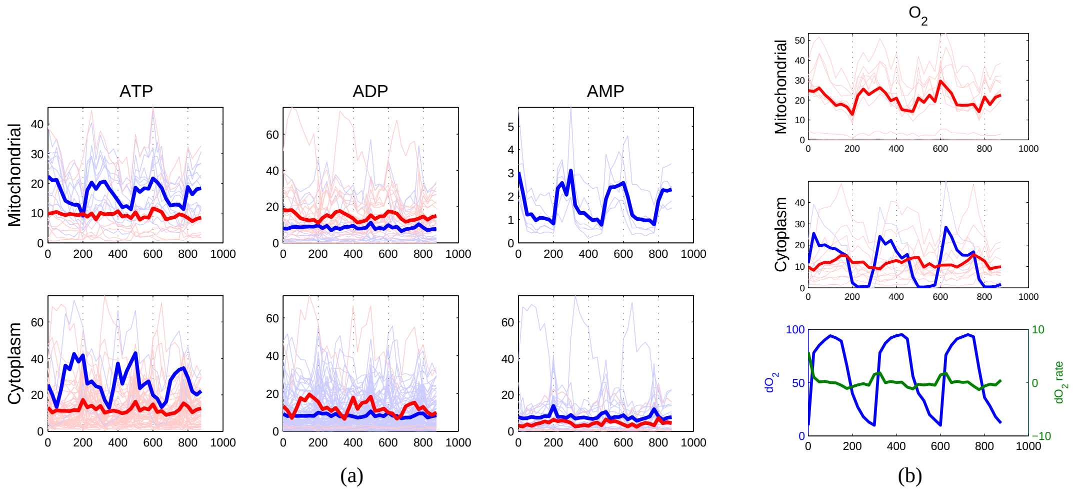

Figure S10: **Enzymatic genes for ATP/ADP/AMP and O_2 reactions**. (a): Time courses of the expression levels for the enzymatic genes catalyzing reactions involving ATP, ADP and AMP. The reactions are subdivided in mitochondrial and cytoplasmic ("cytoplasm" referring to the entire cell with the exclusion of the mitochondria) compartments and according on whether the metabolite is to be considered a substrate (red line) or a product (blue line) of the reaction. Thick curves represent the average over the mRNA expression of the corresponding enzymes. Information abound compartment and reaction direction is extrapolated from [17]. The expression of the enzymatic genes is taken as a measure of the flux of metabolites through the reaction node (scales are however not indicative). The peaks of consumption of ATP in the cytoplasm in correspondence of the main bursts are small but visible. More visible is the pattern of ATP-producing enzymes in both compartments. In the cytoplasm this is essentially due to the pyruvate kinase enzyme Cdc19 transforming phosphoenolpyruvate into pyruvate during anaerobic respiration, while in the mitochondria it is due to the oxidative phosphorylation pathway. The fermentative recharging of ATP in the cytoplasm is quite in antiphase with the respiratory mitochondrial one (scale here can be even misleading: aerobic ATP production is of course far more efficient than anaerobic one). Notice that during the bursts of transcription, ATP hydrolysis rather than peaks of ADP induces peaks in the production of AMP, as is expected for high energy demanding reactions such as RNA polynucleotide synthesis. (b): Time course of expression for enzymes of reactions involving O_2. Color, line thickness and compartment subdivision is the same as above. The third plot is the trace of dissolved O_2 (blue line, data reproduced from [1]), and the dO_2 ratio (green line). Its trend follows closely the cytoplasmic "oxygen production" (blue curve in the middle plot), which essentially is the time course of the catalases, enzymes detoxifying reactive oxygen species such as H_2O_2. Qualitatively the main discrepancy between the two curves occurs in the 50 min interval following the bursts (e.g. $200 \div 250$ min.) where dO_2 keeps decreasing while the concentration of the Catalases mRNAs remains basically at zero level. From the top plot, the explanation could be that this is the interval in which mitochondrial respiration starts, thereby consuming O_2.
